# 

**Published:** 1903-02

**Authors:** 


### CONTENTS.

ORIGINAL ARTICLES:
- Trypanosomes, With Special Reference to Surra. By WALTER G. BOWERS . . . . 65
- President's Address. By WILLIAM HERBERT LOWE, D.V.S. . . . . . . . . . 71
- Vitality and Immunity. By CARL SCHULIN, M.D. . . . . . . . . . . 77
- Hemorrhagic Septicæmia. By M. H. REYNOLDS, M.D., V.M. . . . . . . . . 88
- Persistency of the Urachus. By S H. SWAIN, V.S. . . . . . . . . . 95
- The Foot of the Horse. By L. L. CONKEY, D.V.S. . . . . . . . . . . 97

ABSTRACTS FROM FOREIGN JOURNALS . . . . . . . . . . . . 102

REPORTS OF CASES:
- Rupture of Rectum and Vagina in a Mare During Parturition: Successful Treatment. By S. H. BAUMAN, D V.S. . . . . . . . . . . . . . . . . . 104
- Obstruction of Bowels in a Horse, Due to Abscess in the Lumbosacral Region. By S. H. BAUMAN, D.V S. . . . . . . . . . . . . . . . 105
- Embolism of the Pulmonary Artery in a Horse. By J. W. SCOTT, V.S. . . . . . 106
- Five Cases of Azoturia. By A. S. BRODIE, V.S. . . . . . . . . . . 109
- History of a Case of Tetanus. By R. BARNES, V.S. . . . . . . . . . 110
- Report of a Case of Fungosus Toxicum Paralyticus. By W. A. SWAIN, V.S. . . . . 112

EDITORIALS . . . . . . . . . . . . . . . . . 114

NECROLOGY . . . . . . . . . . . . . . . . . 115

ABSTRACT OF REPORT OF THE STATE VETERINARY SURGEON OF MONTANA . . 116

NEW JERSEY ECHOES—SOCIETY NOTES—SOCIETY PROCEEDINGS—PERSONALS . 122-131

